# Uncoupling substrate delivery from export gate activation reveals distinct roles of the flagellar ATPase complex

**DOI:** 10.3389/fmicb.2026.1841110

**Published:** 2026-05-21

**Authors:** Tohru Minamino, Miki Kinoshita, Yuki Tajimi, Takayuki Uchihashi, Keiichi Namba

**Affiliations:** 1 Graduate School of Frontier Biosciences, The University of Osaka, Suita, Osaka, Japan; 2 JEOL YOKOGUSHI Research Alliance Laboratories, The University of Osaka, Suita, Osaka, Japan; 3 Department of Physics, Nagoya University, Nagoya, Aichi, Japan; 4 Institute for Glyco-Core Research (IGCORE), Nagoya University, Nagoya, Aichi, Japan; 5 Quantum-Based Frontier Research Hub for Industry Development (Q-BReD), Nagoya University, Nagoya, Aichi, Japan; 6 Exploratory Research Center on Life and Living Systems, National Institutes of Natural Sciences, Okazaki, Aichi, Japan

**Keywords:** ATPase complex, flagellar type III secretion system, FliH, Flii, FliJ, ion coupling, protein export

## Abstract

The bacterial flagellar type III secretion system (fT3SS) exports structural subunits required for flagellar assembly by coupling protein translocation to ion motive force across the cytoplasmic membrane. Efficient activation of the transmembrane export gate depends on a cytoplasmic ATPase complex composed of FliH, FliI, and FliJ, which are also involved in substrate delivery. However, how these proteins mechanistically integrate substrate delivery with gate activation remains unclear. Here, we uncoupled these two functions by cross-complementation analyses using ATPase components from the Na^+^-driven polar flagellum of *Vibrio* and the H^+^-driven flagellum of *Salmonella*. Despite low sequence identity, *Vibrio* FliJ complemented a *Salmonella* Δ*fliJ* mutant and restored Na^+^-independent protein export to a substantial extent, demonstrating a highly conserved mechanism of export gate activation. In contrast, *Vibrio* FliH and FliI exhibited interspecies incompatibility when expressed individually, and their co-expression in a *Salmonella* Δ*fliH-fliI* mutant supported protein export only under Na^+^-coupled conditions, consistent with the failure to activate the H^+^-driven export gate. Biochemical analyses revealed species-specific interactions between FliH and FliI, while high-speed atomic force microscopy showed that the *Vibrio* FliH-FliI complex retains the ability to assemble into ring-shaped structures. Together, these findings demonstrate that ATPase ring assembly and substrate delivery are mechanistically separable from export gate activation, revealing distinct and differentially conserved roles of the flagellar ATPase complex in coupling ATP hydrolysis to ion-driven protein export.

## Introduction

**Figure 1:**
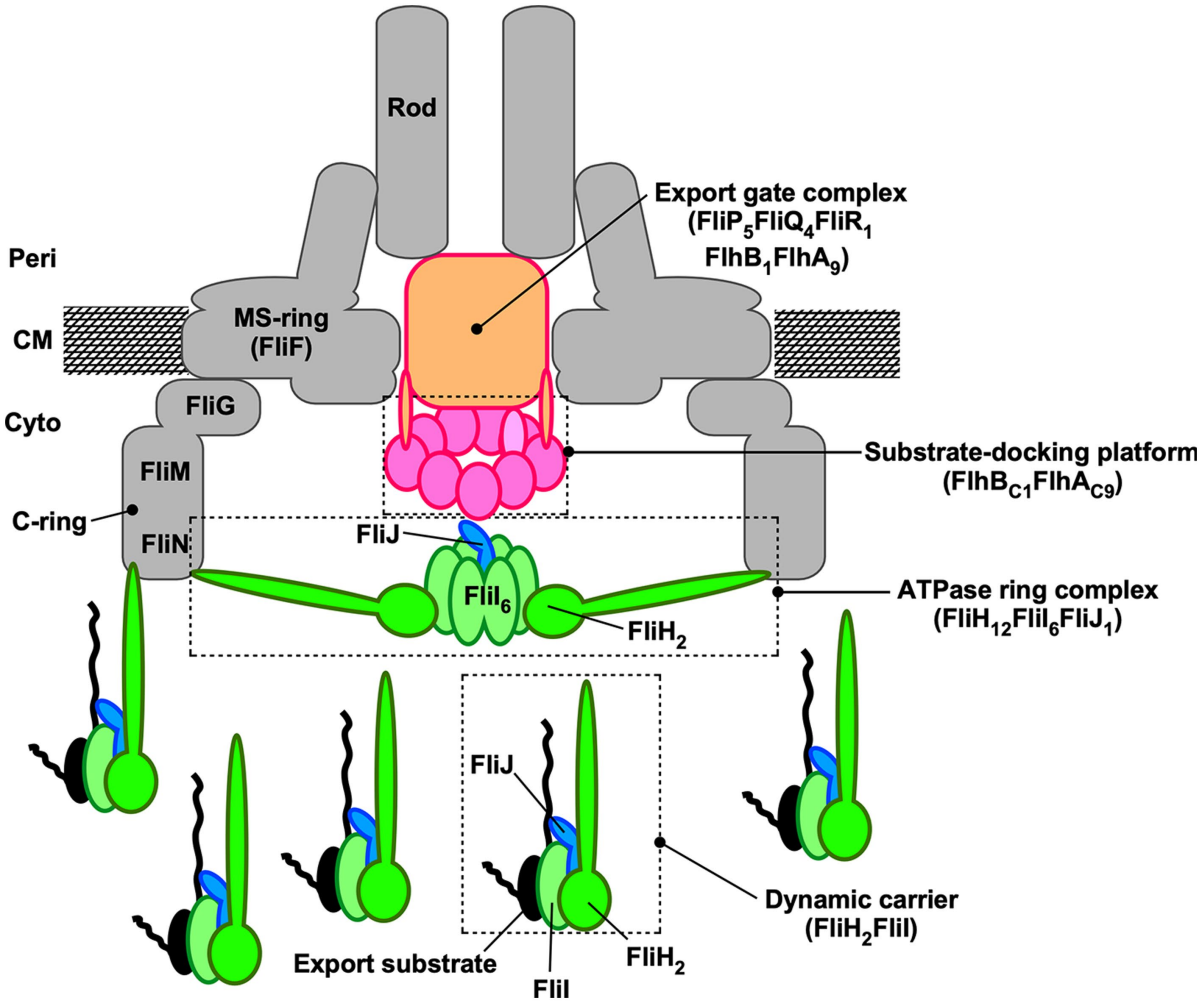
Schematic diagram of the flagellar type III secretion system. The flagellar type III secretion system (fT3SS) is composed of a transmembrane export gate complex with a stoichiometry of nine FlhA subunits, one FlhB subunit, five FliP subunits, four FliQ subunits, and one FliR subunit, and a cytoplasmic ATPase ring complex composed of 12 FliH subunits, six FliI subunits, and one FliJ subunit. FliH and FliI also form a heterotrimeric FliH_2_-FliI complex in the cytoplasm. The export gate complex resides within the central pore of the basal body MS ring and functions as an ion-driven protein transporter. The C-terminal domains of FlhA and FlhB project into the central cavity of the C-ring and serve as a substrate-docking platform. The cytoplasmic ATPase ring complex associates firmly with the basal body C-ring and serves as an ATP-driven activator that enables the export gate complex to operate as a highly efficient H^+^-driven protein transporter. The FliH_2_-FliI complex functions as a dynamic carrier that delivers FliJ and export substrates from the cytoplasm to the substrate-docking platform. Peri, periplasm; CM, cytoplasmic membrane; Cyto, cytoplasm.

The bacterial flagellum is a supramolecular motility machine that allows bacterial cells to migrate toward more favorable environments. It consists of at least three distinct functional parts: the basal body, which functions as a rotary motor powered by ion motive force across the cytoplasmic membrane; the filament, which acts as a helical propeller to generate thrust; and the hook, which functions as a universal joint connecting the basal body and filament. For construction of the flagellum beyond the cell membranes, flagellar structural subunits are transported by the flagellar type III secretion system (fT3SS) located at the base of the flagellum and assemble at the distal end of the growing structure. The fT3SS consists of a transmembrane export gate complex composed of FlhA, FlhB, FliP, FliQ, and FliR, and a cytoplasmic ATPase complex composed of FliH, FliI, and FliJ (Figure 1) (Minamino and Kinoshita, 2023). These component proteins share sequence and functional similarities with the virulence-associated type III secretion system, also known as the injectosome. This system delivers various effector proteins directly into eukaryotic host cells during the infection process (Wagner and Diepold, 2020).

In recent years, significant progress has been made in understanding the structural and functional basis of the transmembrane export gate complex (Minamino et al., 2022a). The export gate is embedded within a central pore of the basal body MS-ring (Johnson et al., 2021). FliP, FliQ, and FliR form a protein-export channel that allows export substrates to be translocated across the cytoplasmic membrane (Kuhlen et al., 2018; Kinoshita et al., 2025). FlhB associates with the FliPQR complex (Kuhlen et al., 2020) and regulates dynamic opening and closing of the cytoplasmic gate (Kinoshita et al., 2021). FlhA surrounds the FliPQR-FlhB complex (Morimoto et al., 2014) and serves as a dual-ion channel that conducts proton (H^+^) and sodium ion (Na^+^) (Minamino et al., 2016b). Thus, the export gate functions as an ion-protein antiporter that couples inward-directed ion flow with outward-directed protein transport.

The C-terminal cytoplasmic of FlhA (FlhA_C_) forms a nonameric ring structure that projects into the central cavity of the basal body C-ring (Abrusci et al., 2013). Structural and biochemical analyses have revealed that flagellar export chaperones and their substrates dock onto this FlhA ring structure prior to secretion, providing a mechanistic basis for substrate recognition and ordered export (Bange et al., 2010; Kinoshita et al., 2013; Xing et al., 2018).

The cytoplasmic ATPase ring complex is composed of 12 copies of FliH, six copies of FliI, and a single copy of FliJ (Minamino et al., 2022b) and functions as an ATP-dependent activator that converts the export gate complex into a highly efficient H^+^-driven protein transporter (Minamino et al., 2014). This complex is structurally similar to the cytoplasmic portions of F_O_F_1_-ATP synthase and V_O_V_1_-ATPase (Imada et al., 2007; Ibuki et al., 2011; Imada et al., 2016). FliI forms a homo-hexamer that hydrolyzes ATP at subunit interfaces (Claret et al., 2003; Kazetani et al., 2009), while FliJ penetrates the central pore of the FliI_6_ ring and acts as a central stalk that activates the export gate through interaction with FlhA (Ibuki et al., 2013). FliH forms a homodimer that functions as a peripheral stalk, anchoring the FliI_6_-FliJ ring complex to C-ring via interactions with FliN (Minamino and Macnab, 2000a; González-Pedrajo et al., 2002; González-Pedrajo et al., 2006; McMurry et al., 2006; Paul et al., 2006; Minamino et al., 2009; Hara et al., 2012; Notti et al., 2015). Furthermore, recent structural studies of type III ATPases have highlighted conserved features of hexameric ATPase assemblies and their interactions with central stalk proteins such as FliJ, suggesting a common mechanism of energy coupling across secretion systems (Majewski et al., 2019).

When the ATPase ring complex is functional, the export gate preferentially uses proton motive force over a wide range of environmental conditions (Minamino and Namba, 2008; Paul et al., 2008; Minamino et al., 2011). In contrast, when the ATPase complex is non-functional, the export gate switches to Na^+^-driven export (Minamino et al., 2016b; Minamino et al., 2021a). Moreover, when the membrane potential exceeds a critical threshold, an otherwise inactive export gate complex autonomously converts into a H^+^-driven transporter even in the absence of external Na^+^ (Minamino et al., 2021b). These observations suggest that ATP hydrolysis by the ATPase complex switches the export gate from a dual-fuel mode to a highly efficient H^+^-driven mode; however, the molecular mechanism underlying this functional coupling remains unclear.

In addition to forming the ring complex for gate activation, FliH and FliI form a heterotrimeric FliH_2_-FliI complex diffusing around in the cytoplasm (Minamino and Macnab, 2000a; Auvray et al., 2002; Bai et al., 2014). This complex is thought to act as a dynamic carrier that delivers FliJ, export substrates, and chaperone-substrate complexes to the substrate-docking platform, thereby ensuring the strict order of flagellar assembly (Minamino and Macnab, 2000a; Auvray et al., 2002; Thomas et al., 2004; Imada et al., 2010; Minamino et al., 2012; Minamino et al., 2016a; Inoue et al., 2018; Rossi et al., 2023; Kinoshita et al., 2024). Thus, the cytoplasmic ATPase complex appears to play at least two mechanistically distinct roles in flagellar protein export: activation of the export gate and delivery of export substrates. How these two functions are coordinated, and to what extent they can be mechanistically separated, remains unknown.

To address these questions, we performed cross-complementation analyses using ATPase components from the fT3SSs of the Na^+^-driven polar flagellum of *Vibrio alginolyticus* (hereafter referred to as *Vibrio*) and the H^+^-driven flagellum of *Salmonella enterica* serovar Typhimurium (hereafter referred to as *Salmonella*). We show that *Vibrio* FliJ is functional within the *Salmonella* fT3SS in the presence of native FliH and FliI, whereas *Vibrio* FliH and FliI exhibit interspecies incompatibility when expressed individually. Co-expression of *Vibrio* FliH and FliI partially restores function, and high-speed atomic force microscopy reveals that the *Vibrio* FliH-FliI complex retains the ability to assemble into ring-like structures. These findings reveal evolutionary constraints on the functional integration of the flagellar ATPase complex and provide insights into the mechanistic separation of its roles in gate activation and substrate delivery.

## Materials and methods

### *Salmonella* strains, plasmids, DNA manipulations, and media

*Salmonella* strains and plasmids used in this study are listed in Table 1. DNA manipulations were performed using standard protocols. The cloned DNA fragments were confirmed by DNA sequencing (Eurofins Genomics). L-broth contained 1% (w/v) tryptone, 0.5% (w/v) yeast extract, and 0.5% (w/v) NaCl. Soft tryptone agar plates contained 1% (w/v) tryptone, 0.5% (w/v) NaCl, and 0.35% (w/v) agar. Ampicillin was added as needed at a final concentration of 100 μg ml^−1^.

**Table 1:** *Salmonella* strains and plasmids used in this study.

Strain/Plasmid	Relevant characteristics	References
*Salmonella*		
SJW1368	∆(*cheW-flhD*); master operon mutant	Ohnishi et al. (1994)
MKM11	∆*fliH*	González-Pedrajo et al. (2002)
MKM30	∆*fliI*	Minamino et al. (2006)
MKM40	∆*fliJ*	Minamino et al. (2009)
MMHI001	∆*fliH-fliI*	Minamino et al. (2006)
MMHIJ001	∆*fliH-fliI-fliJ*	Minamino et al. (2011)
MMHIJ0117	∆*fliH-fliI-fliJ flhB(P28T)*	Minamino et al. (2011)
Plasmids		
pTrc99A	Expression vector	GE Healthcare
pTrc99AFF4	Expression vector	Ohnishi et al. (1997)
pMM309	pTrc99AFF4/StFliH	Minamino and Macnab (2000a)
pMM404	pTrc99AFF4/StFliJ	Minamino et al. (2000)
pMM1702	pTrc99A/His-StFliI	Minamino and Macnab (2000b)
pMM1719	pTrc990117AFF4/StFliI	Minamino and Macnab (2000a)
pMMHI001	pTrc99AFF4/ StFliH + StFliI	Minamino and Namba (2008)
pMKM1702iH	pTrc99A/ His-StFliI + StFliH	Imada et al. (2016)
pMKM1702Va	pTrc99A/His-VaFliI	This study
pMKM2001Va	pTrc99AFF4/VaFliH	This study
pMKM2002Va	pTrc99AFF4/VaFliI	This study
pMKM2003Va	pTrc99AFF4/VaFliJ	This study
pMKM2004Va	pTrc99AFF4/VaFliH + VaFliI	This study
pMKM2005Va	pTrc99A/ His-StFliI + VaFliH	This study
pMKM2006Va	pTrc99A/ His-VaFliI + StFliH	This study
pMKM2007Va	pTrc99A/ His-VaFliI + VaFliH	This study

#### Sequence alignment

Sequence alignment was carried out using CLUSTAL-*Ω*^1^.

#### Structural modeling

Structural models were generated using the AlphaFold3 prediction server^2^. Structural comparisons between *Salmonella* and *Vibrio* flagellar ATPase components were conducted by UCSF ChimeraX (Pettersen et al., 2021). All figures including structural models of the flagellar ATPase components were prepared using UCSF ChimeraX.

### Soft-agar motility assays

Fresh colonies were inoculated onto soft agar plates supplemented with ampicillin and incubated at 30 °C. The assay was performed at least seven times to confirm the reproducibility of the results.

### Secretion assays

*Salmonella* cells were grown in 5 mL L-broth supplemented with ampicillin at 30 °C with shaking until the cell density reached an OD_600_ of approximately 1.2–1.4. Cultures were then centrifuged to separate cell pellets and culture supernatants. Proteins in the whole-cell and culture supernatant fractions were normalized to the OD_600_ of each culture to ensure equivalent cell numbers.

Cell pellets were resuspended directly in SDS loading buffer [62.5 mM Tris–HCl (pH 6.8), 2% (w/v) sodium dodecyl sulfate (SDS), 10% (w/v) glycerol, and 0.001% (w/v) bromophenol blue] supplemented with 1 μL of 2-mercaptoethanol. Proteins in the culture supernatants were precipitated with 10% (w/v) trichloroacetic acid on ice for 1 h, centrifuged (20,000 g, 20 min, 4 °C), and resuspended in Tris-SDS loading buffer (one volume of 1 M Tris–HCl mixed with nine volumes of SDS loading buffer) containing 1 μL of 2-mercaptoethanol.

After heating at 95 °C for 3 min, protein samples were separated by SDS-polyacrylamide gel electrophoresis (SDS-PAGE) and subjected to immunoblotting using polyclonal anti-FlgD antibody. Immunoblotting was performed with an iBind Flex Western Device according to the manufacturer’s instructions (Thermo Fisher Scientific). Chemiluminescent signals were detected using Amersham ECL Prime Western Blotting Detection Reagent (Cytiva) and captured with a Luminoimage Analyzer LAS-3000 (GE Healthcare). Image data were processed using Photoshop software (Adobe). The assay was performed at least three times to confirm the reproducibility of the results.

### Pull-down assays by Ni-affinity chromatography

The *Salmonella* SJW1368 strain carrying pTrc99A-based plasmids co-expressing of untagged FliH and His-FliI was grown overnight at 30 °C in 100 mL L-broth supplemented with ampicillin. Cells were harvested by centrifugation, suspended in 25 mL of TN buffer [20 mM Tris–HCl (pH 8.0), 500 mM NaCl] containing 25 mM imidazole, and disrupted by sonication. After centrifugation to remove undisrupted cells and cell debris, the clarified cell lysates were loaded onto a nickel-nitrilotriacetic acid (Ni-NTA) agarose column (QIAGEN). After washing the column with the TN buffer containing 50 mM imidazole, bound proteins were eluted with the same buffer containing increasing concentrations of imidazole (100, 250, and 500 mM) in a stepwise manner. Eluted fractions were analyzed by SDS-PAGE, followed by Coomassie Brilliant Blue (CBB) staining.

### Size exclusion chromatography

*Salmonella* FliH-His-FliI complex, *Vibrio* His-FliH, and *Vibrio* FliH-His-FliI complex, purified by Ni-affinity chromatography, were applied to a Superdex 200 16/60 column (GE Healthcare) equilibrated with buffer containing 50 mM Tri-HCl (pH 8.0), 150 mM NaCl and 1 mM EDTA at a flow rate of 1.0 mL min^−1^. Elution fractions were analyzed by SDS-PAGE, followed by CBB staining.

Analytical size exclusion chromatography was performed with a Superdex 200 Increase 10/300 column (GE Healthcare). Purified samples were applied to the column equilibrated with buffer containing 50 mM Tris–HCl (pH 8.0), 150 mM NaCl, 1 mM EDTA, and 1 mM DTT at a flow rate of 0.5 mL min^−1^. Unless otherwise stated, size exclusion chromatography was performed in the absence of nucleotide.

### Chemical cross-linking

Purified protein samples were dialyzed against 20 mM sodium phosphate buffer (pH 7.0) containing 100 mM NaCl at 4 °C, and then glutaraldehyde was added to 50 μL aliquots of each protein sample (10 μM final protein concentration) to a final concentration of 0.1% (v/v). The cross-linking reaction was allowed to proceed for 30 min at room temperature and was quenched by the addition of Tris–HCl to a final concentration of 100 mM. Following quenching, 60 μL of 2 × SDS loading buffer [125 mM Tris–HCl (pH 6.8), 4% (w/v) SDS, 20% (w/v) glycerol, and 0.002% (w/v) bromophenol blue] supplemented with 1 μL of 2-mercaptoethanol was added to each sample. Samples were heated at 95 °C for 5 min, and proteins were subsequently separated by SDS-PAGE using a 10–20% gradient polyacrylamide gel and analyzed by CBB staining and immunoblotting using anti-His-tag mAb-HRP-DirecT (MBL, Japan). All image data were processed using Photoshop software (Adobe). Each experiment was performed at least three times independently.

### High-speed atomic force microscopy

High-speed atomic force microscopy (HS-AFM) imaging was performed in solution using a laboratory-built HS-AFM system (Ando et al., 2001; Ando et al., 2008). The purified FliH-FliI complex was diluted to a final concentration of 2 μM in observation buffer containing 25 mM Tris–HCl (pH 8.0), 75 mM NaCl, 5 mM MgCl_2_, and 5 mM ADP-AlF_4_^−^ (a transition-state analog). After incubation at room temperature for a few minutes, a 2 μL aliquot was deposited onto a freshly cleaved mica surface and incubated for 5 min. Unbound molecules were removed by washing the surface with 100 μL of the observation buffer. HS-AFM imaging was performed in 70 μL of the observation buffer at room temperature.

HS-AFM imaging was carried out in tapping mode using small cantilevers (AC7, Olympus) with a resonant frequency of approximately 800 kHz and a spring constant of approximately 0.2 N/m. An amorphous carbon pillar was grown on the cantilever tip by electron beam deposition (EBD) and sharpened by argon gas plasma etching to serve as a probe. HS-AFM images were processed by applying a low-pass filter to reduce random noise and a line-by-line background subtraction filter to correct the xy-plane background using a laboratory-built Python-based viewer software (pyNuD).

## Results

### Sequence and structural comparison of FliH, FliI, and FliJ

To assess the degree of conservation between the flagellar ATPase components of *Vibrio* and *Salmonella*, we first compared the amino acid sequences of FliH, FliI, and FliJ. Pairwise sequence alignments revealed that *Vibrio* FliI is relatively well conserved, showing 56.2% identity and 68.2% similarity to *Salmonella* FliI, whereas *Vibrio* FliH and FliJ exhibit much lower sequence identity (21 and 18%, respectively), despite moderate similarity (38.1 and 49.3%, respectively) (Supplementary Figures 1A–3A).

To examine whether these sequence differences translate into major structural divergence, we compared the available crystal structures of the *Salmonella* FliH-FliI complex (PDB ID: 5B0O) and *Salmonella* FliJ (PDB ID: 3AJW) with AlphaFold-predicted structures of their *Vibrio* homologs. Despite the low sequence identity, the predicted structures of *Vibrio* FliH, FliI, and FliJ closely resemble the corresponding *Salmonella* crystal structures, exhibiting highly conserved overall folds (Supplementary Figures 1B–3B).

These observations indicate that although the primary sequences of FliH and FliJ have diverged substantially between *Vibrio* and *Salmonella*, their overall structures remain highly conserved. This raises the possibility that species-specific incompatibility arises not from global structural differences but from subtle variations at protein–protein interaction interfaces, and this hypothesis is tested in the functional analyses described below.

### Functional compatibility of *Vibrio* FliH, FliI, and FliJ in *Salmonella* fT3SS

To examine the functional compatibility of the *Vibrio* ATPase components with those of *Salmonella* fT3SS, we performed cross-complementation assays using *Salmonella* Δ*fliH*, Δ*fliI*, and Δ*fliJ* mutant strains expressing the corresponding *Vibrio* proteins. Flagella-driven motility and fT3SS-mediated protein secretion were assessed by soft-agar motility assays and immunoblotting using a polyclonal antibody against FlgD, a representative export substrate of the fT3SS, respectively.

**Figure 2:**
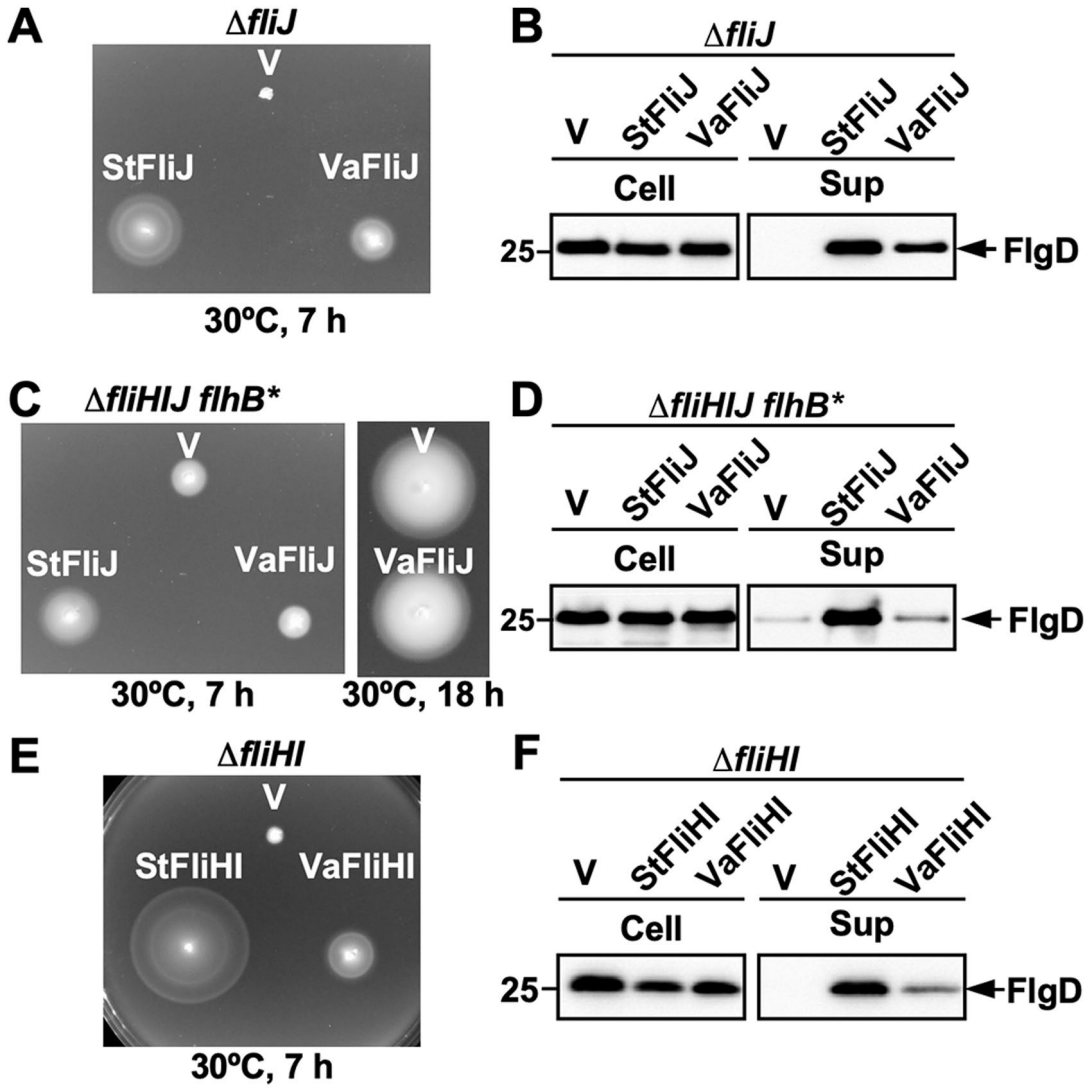
Cross-complementation analysis of *Vibrio* ATPase components in *Salmonella*. **(A)** Soft-agar motility assays of a *Salmonella enterica ser*ovar Typhimurium Δ*fliJ* mutant carrying pTrc99AFF4 (vector control; V), pMM404 (*St*FliJ), or pMKM2003Va (*Va*FliJ). Plates were incubated at 30 °C for 7 h. At least seven independent assays were performed. **(B)** Secretion assays analyzed by immunoblotting using a polyclonal antibody against the hook-capping protein FlgD. Whole-cell (Cell) and culture supernatant (Sup) fractions were prepared from the transformants shown in **(A)**. A 25-kDa molecular mass marker is shown on the left. At least three independent assays were performed. **(C)** Soft-agar motility assays of a *Salmonella Δ*fl*iH-fliI-fliJ flhB*(P28T) mutant (ΔfliHIJ flhB*) carrying pTrc99AFF4 (V), pMM404 (*St*FliJ), or pMKM2003Va (*Va*FliJ). **(D)** Secretion assays analyzed by immunoblotting using a polyclonal anti-FlgD antibody. Whole-cell and culture supernatant fractions were prepared from the transformants shown in **(C)**. **(E)** Soft-agar motility assays of a *Salmonella Δ*fl*iH-fliI* mutant (Δ*fliHI*) carrying pTrc99AFF4 (V), pMMHI001 (*St*FliHI), or pMKM2004Va (*Va*FliHI). **(F)** Secretion assays analyzed by immunoblotting using a polyclonal anti-FlgD antibody. Whole-cell and culture supernatant fractions were prepared from the transformants shown in **(E)**.

Expression of *Vibrio* FliJ substantially restored motility (Figure 2A and Supplementary Figure 4) and flagellar protein export (Figure 2B) in the *Salmonella* Δ*fliJ* mutant, indicating that *Vibrio* FliJ is functionally compatible with the *Salmonella* fT3SS. In contrast, expression of *Vibrio* FliH or *Vibrio* FliI alone failed to rescue the motility defects of the *Salmonella* Δ*fliH* or Δ*fliI* mutants, respectively (Supplementary Figure 4), demonstrating that these components are not individually interchangeable between the two species.

We next tested whether the function of *Vibrio* FliJ depends on the presence of *Salmonella* FliH and FliI. Because the *flhB*(P28T) mutation substantially increases the probability of flagellar formation in the absence of FliH and FliI (Minamino and Namba, 2008; Minamino et al., 2011), we analyzed both a *Salmonella* Δ*fliH-fliI-fliJ* mutant and a Δ*fliH-fliI-fliJ flhB*(P28T) mutant. In a *Salmonella* background lacking both FliH and FliI, *Vibrio* FliJ failed to restore motility (Figure 2C and Supplementary Figure 5) or flagellar protein export (Figure 2D), indicating that FliJ-mediated activation of the export gate requires the native *Salmonella* FliH-FliI complex.

Because *Vibrio* FliH exhibits much lower sequence identity to *Salmonella* FliH than for FliI, we next investigated whether *Vibrio* FliH and FliI together can function as the flagellar ATPase complex in the *Salmonella* fT3SS. Simultaneous expression of *Vibrio* FliH and FliI in a *Salmonella* Δ*fliH*-*fliI* mutant partially restored motility (Figure 2E) and protein export activity (Figure 2F). This result suggests that the *Vibrio* FliH-FliI complex can function as a cytoplasmic ATPase complex together with native FliJ in *Salmonella* but is less efficient at activating the transmembrane export gate than the native *Salmonella* FliH-FliI complex.

Together, these results reveal that while *Vibrio* FliJ is compatible with the *Salmonella* export system, functional integration of *Vibrio* FliH and FliI requires their co-expression and remains incomplete, highlighting species-specific constraints in ATPase-export gate coupling.

### 3.3 *Vibrio* FliJ activates the H^+^-driven export gate of *Salmonella* fT3SS independently of Na^+^

The transmembrane export gate complex intrinsically functions as a dual-fuel export engine that exploits both H^+^ and Na^+^ as coupling ions to drive flagellar protein export (Minamino et al., 2016b). In the absence of FliH and FliI, however, the export gate preferentially utilizes the sodium motive force across the cytoplasmic membrane over a wide range of external pH values (Minamino et al., 2016b, 2021a). As a result, *Salmonella* strains lacking FliH and FliI display weak but significant motility in the presence of Na^+^ but are essentially non-motile in its absence (Supplementary Figure 6). Once ATP is hydrolyzed by the FliI_6_ ring complex, FlhA turns an inefficient dual-fuel engine into a highly efficient H^+^-driven export engine through the FliJ-FlhA interaction (Minamino et al., 2011; Minamino et al., 2014). Consequently, the export gate preferentially uses proton motive force as its primary energy source (Minamino et al., 2016b).

**Figure 3:**
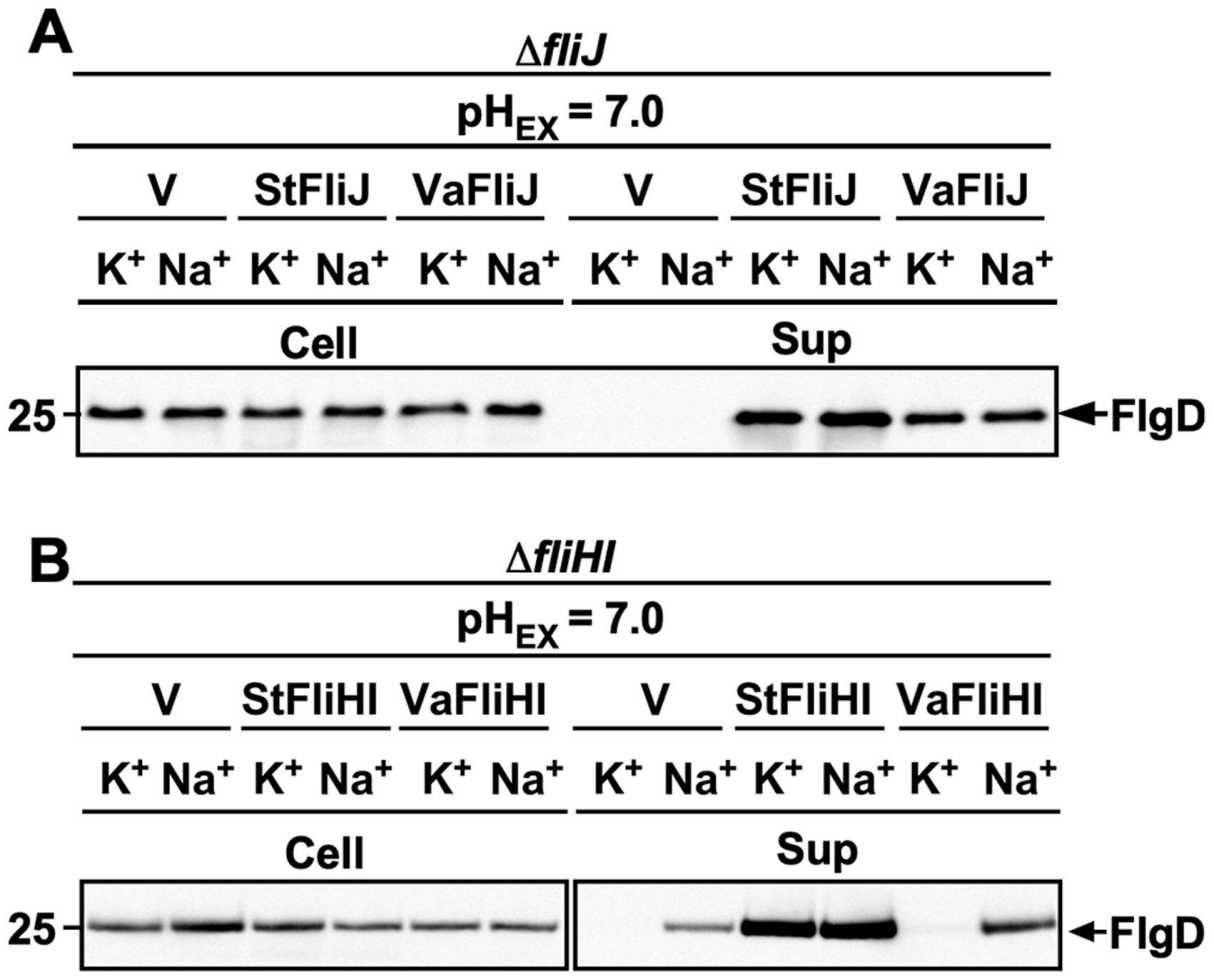
Effect of sodium ions on flagellar protein export in the presence of *Vibrio* ATPase components. Secretion assays analyzed by immunoblotting using a polyclonal anti-FlgD antibody of whole-cell (Cell) and culture supernatant (Sup) fractions prepared from **(A)** a *Salmonella enterica ser*ovar Typhimurium Δ*fliJ* mutant carrying pTrc99AFF4 (vector control; V), pMM404 (StFliJ), or pMKM2004Va (VaFliJ), and **(B)** a *Salmonella enterica ser*ovar Typhimurium Δ*fliHI* mutant carrying pTrc99AFF4 (vector control; V), pMMHI001 (StFliHI), or pMKM2004Va (VaFliHI). Cells were grown exponentially at 30 °C in T-broth (pH 7.0) supplemented with either 100 mM KCl (K^+^) or 100 mM NaCl (Na^+^). The 25-kDa molecular mass marker is shown on the left. At least three independent assays were performed.

To examine whether *Vibrio* FliJ can activate the *Salmonella* export gate in a manner comparable to native FliJ, we analyzed the Na^+^ dependence of flagellar protein export in a *Salmonella* Δ*fliJ* mutant expressing *Vibrio* FliJ. Export of the hook-capping protein FlgD was monitored in the presence of either 100 mM Na^+^ or 100 mM K^+^ (Figure 3A).

In the absence of FliJ, the export gate remains inactive even in the presence of Na^+^ (Supplementary Figure 6), and no FlgD export therefore occurred, consistent with previous observations (Minamino et al., 2016b, 2021a). In contrast, expression of *Vibrio* FliJ restored robust FlgD export regardless of Na^+^ availability, closely resembling the phenotype observed upon expression of native *Salmonella* FliJ (Figure 3A).

These results demonstrate that *Vibrio* FliJ efficiently activates the H^+^-driven export mode of the *Salmonella* flagellar export gate. Thus, despite low sequence identity, FliJ retains a conserved ability to couple the ATPase complex to gate activation across species boundaries.

### 3.4 Species-specific interaction between FliH and FliI underlies interspecies incompatibility

**Figure 4:**
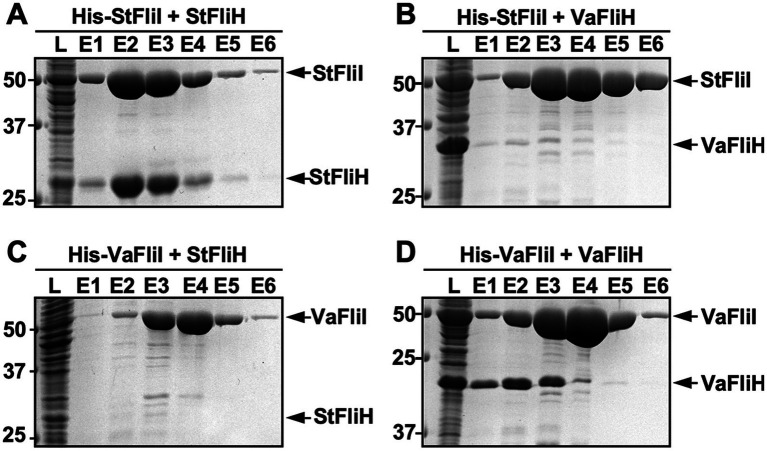
Species-specific interactions between FliH and FliI revealed by pull-down assays. Cell lysates (L) prepared from *Salmonella enterica ser*ovar Typhimurium strain SJW1368 (Δ*cheW–flhD*) harboring plasmids encoding the following combinations were subjected to Ni-NTA affinity chromatography: **(A)** His-tagged *Salmonella* FliI (His-StFliI) with untagged *Salmonella* FliH (StFliH); **(B)** His-StFliI with untagged *Vibrio alginolyticus* FliH (*Va*FliH); **(C)** His-tagged *Vibrio* FliI (His-*Va*FliI) with StFliH; or **(D)** His-VaFliI with VaFliH. After extensive washing with binding buffer [20 mM Tris–HCl (pH 8.0), 500 mM NaCl] containing 25 mM imidazole, bound proteins were eluted sequentially with binding buffer containing 100 mM imidazole (E1), 250 mM imidazole (E2), and 500 mM imidazole (E3–E5). Eluted fractions were analyzed by SDS-PAGE followed by Coomassie Brilliant Blue staining. Molecular mass markers are shown on the left. Three independent assays were performed.

To determine whether the observed interspecies incompatibility of FliH and FliI reflects impaired physical interactions, we examined protein–protein interactions between *Vibrio* and *Salmonella* ATPase components using pull-down assays by Ni-affinity chromatography. When *Salmonella* His-FliI was used as a bait, it efficiently pulled down untagged *Salmonella* FliH (Figure 4A), in agreement with previous observations (Minamino and Macnab, 2000a; Auvray et al., 2002). In contrast, *Salmonella* His-FliI failed to pull down untagged *Vibrio* FliH under the same experimental condition (Figure 4B). Reciprocally, *Vibrio* His-FliI interacted with untagged *Vibrio* FliH (Figure 4D) but not with untagged *Salmonella* FliH (Figure 4C), indicating that native FliH-FliI interactions are preserved within each species but not across species boundaries.

These results demonstrate that FliH-FliI interactions are species-specific and that incompatibility between *Vibrio* and *Salmonella* ATPase components arises from the failure to form heterologous FliH-FliI complexes. This explains why co-expression of *Vibrio* FliH and FliI is required to partially restore function in the *Salmonella* Δ*fliH-fliI* mutant.

### 3.5 The *Vibrio* FliH-FliI complex functions as a dynamic carrier but fails to activate the H^+^-driven export gate

To assess whether the *Vibrio* FliH-FliI complex can activate the *Salmonella* export gate, we examined the ion dependence of flagellar protein export in the *Salmonella* Δ*fliH-fliI* mutant expressing *Vibrio* FliH and FliI. Export of the hook-capping protein FlgD was analyzed in the presence of either 100 mM Na^+^ or 100 mM K^+^ (Figure 3B).

Expression of native *Salmonella* FliH and FliI restored robust FlgD export independently of Na^+^, as consistent with efficient activation of the H^+^-driven export gate. In contrast, co-expression of *Vibrio* FliH and FliI supported FlgD export only in the presence of Na^+^, but not when Na^+^ was replaced with K^+^ (Figure 3B). These results indicate that the *Vibrio* FliH-FliI complex can support substrate delivery to the export gate as a dynamic carrier but is incapable of activating the H^+^-driven transport mode of the *Salmonella* export gate.

### 3.6 *Vibrio* FliI alone fails to function as a dynamic carrier in *Salmonella* T3SS

Overexpression of *Salmonella* FliI alone can bypass the requirement for FliH to a considerable extent (Minamino et al., 2003; Minamino et al., 2006). We therefore examined whether overexpression of *Vibrio* FliI could similarly restore flagella-driven motility of the *Salmonella* Δ*fliH-fliI* mutant. In contrast to *Salmonella* FliI, overexpression of *Vibrio* FliI failed to restore motility under any conditions tested (Supplementary Figure 7A), indicating that *Vibrio* FliI requires its cognate FliH to exert export activity in the *Salmonella* fT3SS.

We next examined whether *Salmonella* FliI alone can act as the ATP-driven activator of the export gate in the absence of FliH by performing soft-agar motility assays in the presence of either 100 mM Na^+^ or 100 mM K^+^ (Supplementary Figure 7B). Overexpression of *Salmonella* FliI enhanced motility only slightly in the presence of 100 mM K^+^, indicating that FliI alone is insufficient to fully activate the H^+^-driven export gate. In contrast, FliI overexpression substantially improved motility in the presence of 100 mM Na^+^, suggesting that FliI alone may function as the substrate carrier for Na^+^-driven export gate. Notably, deletion of *fliJ* abolished motility in the Δ*fliH-fliI* mutant overexpressing *Salmonella* FliI (Supplementary Figure 7A), indicating that FliJ is required for FliI-mediated motility.

Together, these results suggest that *Salmonella* FliI can function as a dynamic carrier that delivers FliJ and export substrates to the export gate, whereas *Vibrio* FliI alone lacks this capability in the *Salmonella* fT3SS.

### 3.7 Oligomerization state of *Vibrio* FliH and FliH-FliI complex

**Figure 5:**
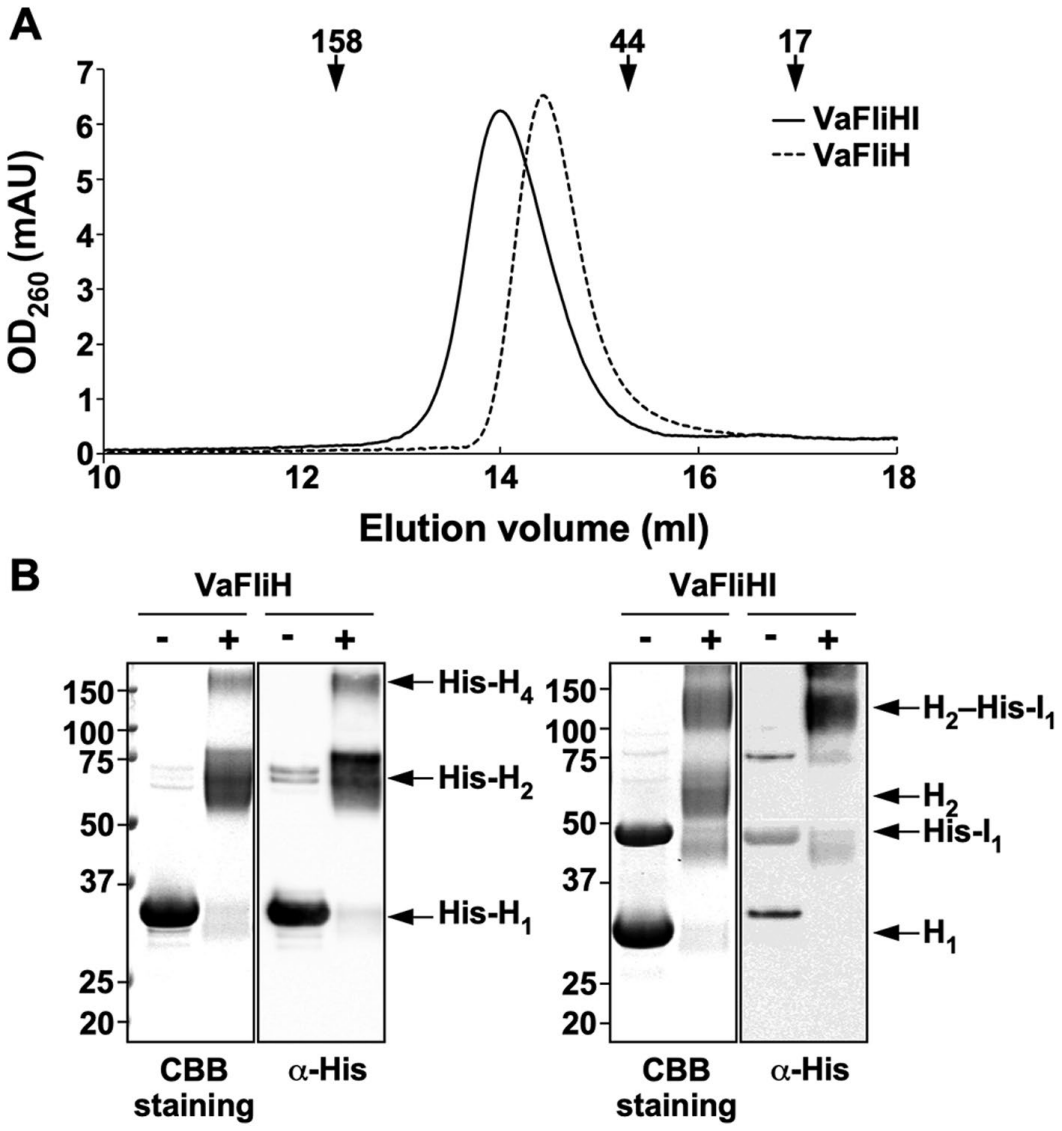
Oligomerization states of the *Vibrio* flagellar ATPase components. **(A)** Analytical size exclusion chromatography. Purified *Vibrio* His-FliH (0.01 mg ml^−1^) and the *Vibrio* FliH-His-FliI complex (0.01 mg ml^−1^) were applied to a Superdex 200 Increase 10/300 column equilibrated with buffer containing 50 mM Tris–HCl (pH 8.0), 150 mM NaCl, 1 mM EDTA, and 1 mM DTT at a flow rate of 0.5 mL min^−1^. Elution peaks of His-*Va*FliH (solid line) and *Va*FliH-His-*Va*FliI (dashed line) were observed at 14.4 and 14.0 mL, respectively. Arrow indicates the elution positions of molecular mass standards (158, 43, and 14 kDa), which eluted at 12.4 mL, 15.3 mL, and 17.0 mL, respectively. **(B)** Chemical cross-linking with glutaraldehyde. Cross-linked products of His-*Va*FliH (left panels) and the *Va*FliH-His-*Va*FliI complex (right panels) were separated by SDS-PAGE and analyzed by CBB staining and immunoblotting using an anti-His antibody. The positions of molecular mass markers (kDa) are indicated on the left. At least three independent assays were performed.

*Salmonella* FliH predominantly forms a homodimer in solution, and *Salmonella* FliH and FliI forms a hetero-trimeric FliH_2_-FliI complex (Minamino and Macnab, 2000a). To determine whether the functional defect of the *Vibrio* FliH-FliI ATPase complex arises from improper assembly, we purified *Vibrio* His-FliH and FliH-His-FliI complex by size exclusion chromatography (Figure 5A) and analyzed their oligomeric states by chemical cross-linking (Figure 5B).

Given that the deduced molecular mass of *Vibrio* His-FliH is approximately 32.6 kDa, chemical cross-linking analysis revealed that purified His-FliH predominantly formed dimers in solution. Moreover, a cross-linked product corresponding to a tetramer was also detected albeit at low frequency. Because no cross-linked product corresponding to a trimer was observed, this tetramer is likely a dimer of the dimer. In contrast, cross-linking of the purified *Vibrio* FliH-His-FliI complex yielded two major species: one corresponding to the FliH dimer and another corresponding to a heterotrimeric FliH_2_-FliI assembly with a deduced molecular mass of approximately 109.3 kDa. These results indicate that the inability of the *Vibrio* FliH-FliI complex to activate the export gate does not arise from gross defects in heterotrimer formation.

### 3.8 *Vibrio* FliH-FliI complex assembles into a ring-shaped structure as visualized by HS-AFM

*Salmonella* FliI efficiently forms a homohexamer in the presence of Mg^2+^ and the non-hydrolysable ATP analog ADP-AlF_4_^−^ (Minamino et al., 2006). Consistently, EscN, a FliI homologue of the virulence-associated type III secretion system, form a homohexamer, and this EscN hexameric structure has been solved by cryo-electron microscopy image analysis (Majewski et al., 2019). AlphaFold predictions suggest that *Vibrio* FliI can form a similar homohexameric structure (Supplementary Figure 8). To examine whether the failure of the *Vibrio* FliH_2_-FliI complex to activate the *Salmonella* export gate results from its defect in higher-order assembly, we purified *Salmonella* and *Vibrio* FliH_2_-FliI heterotrimeric complexes by size exclusion chromatography (Supplementary Figure 9), incubated them with 5 mM Mg^2+^ and 5 mM ADP-AlF_4_^−^, and visualized them by HS-AFM.

**Figure 6:**
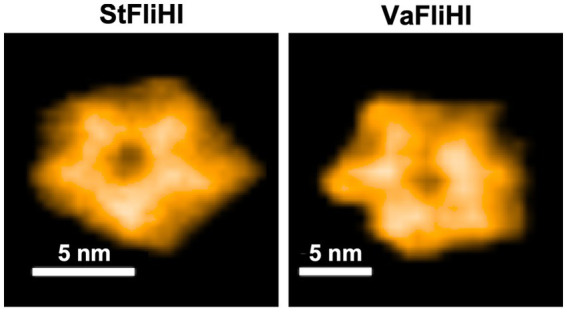
HS-AFM visualization of ring-shaped assemblies formed by the purified FliH-FliI complex. Representative high-speed atomic force microscopy (HS-AFM) images of the *Salmonella* (*St*FliHI, left panel) and *Vibrio* (*Va*FliHI, right panel) FliH-FliI complexes adsorbed onto a mica surface in buffer containing 5 mM Mg^2+^ and 5 mM ADP-AlF_4_^−^. The image of the *St*FliHI complex (left) was cropped from an original image acquired with a scan size of 60 × 60 nm, 150 × 150 pixels, and a frame rate of 2 fps. The image of the *Va*FliHI complex (right) was cropped from an original image acquired with a scan size of 100 × 100 nm, 150 × 150 pixels, and a frame rate of 1 fps.

HS-AFM imaging revealed that the *Vibrio* FliH_2_-FliI complex assembles into a ring-shaped structure on mica surfaces (Figure 6). The overall dimensions and ring morphology were comparable to those observed for the *Salmonella* FliH_2_-FliI complex under the same condition, and this ring-shaped structure was stably maintained during HS-AFM imaging.

Together with the biochemical and functional data, these observations indicate that the *Vibrio* FliH_2_-FliI complex retains the intrinsic capacity to assemble into the ATPase ring-like structure. Thus, formation of the ATPase ring is not sufficient for the activation of the H^+^-driven export gate, highlighting functional separation between ATPase ring assembly, substrate delivery, and export gate activation within the flagellar ATPase complex. It should be noted that the FliI hexameric structures observed in this study were assembled *in vitro* under defined experimental conditions, and their exact structural and functional states *in vivo* remain to be further investigated.

## 4 Discussion

In this study, we dissected the functional integration of the flagellar ATPase complex through cross-complementation between the *Vibrio* and *Salmonella fT3SS*. Our analyses demonstrate that the ATPase complex fulfills at least two mechanistically distinct roles during flagellar protein export: delivery of export substrates to the export gate and activation of the H^+^-driven export engine. While *Vibrio* FliJ was functional within the *Salmonella* fT3SS, the *Vibrio* FliH_2_-FliI complex could only partially substitute for its *Salmonella* counterpart (Figure 2). Despite retaining the ability to assemble into a ring-shaped complex (Figure 6), the *Vibrio* FliH_2_-FliI complex can support only the Na^+^-dependent flagellar protein export but failed to activate the H^+^-driven export engine (Figure 3B). These findings reveal distinct evolutionary constraints acting on the different functions of the flagellar ATPase complex.

One of the most striking observations in this study is the functional compatibility of *Vibrio* FliJ in the *Salmonella* fT3SS. Expression of *Vibrio* FliJ restored Na^+^-independent export of FlgD in a *Salmonella* Δ*fliJ* mutant (Figure 3A), indicating that it can activate the H^+^-driven mode of the export gate. This level of functional conservation is particularly remarkable given the low sequence identity between the two FliJ proteins (Supplementary Figure 3A). Structural comparisons between the *Salmonella* crystal structure and an AlphaFold-predicted model of *Vibrio* FliJ revealed a high degree of structural similarity (Supplementary Figure 3B), suggesting that key interaction surfaces required for export gate activation are strongly conserved. In fact, two highly conserved, surface-exposed residues, Phe-72 and Leu-76 of FliJ, are directly involved in the interaction with FlhA (Supplementary Figure 3A). Mutational analyses have shown that the FliJ(F72A) and FliJ(L76A) variants require the support of FliH and FliI to exert their export function to a considerable degree (Ibuki et al., 2013). These observations suggest that FliJ plays a central role in activating the export gate through its interaction with FlhA. The high cross-species compatibility of FliJ, despite its low sequence identity, further supports the idea that FliJ functions as a universal activator of the export gate and interacts with a structurally conserved and evolutionarily constrained component of the export machinery, most likely the FlhA platform. In contrast, FliH and FliI appear to have co-evolved with species-specific regulatory networks, allowing fine-tuning of export gate activation in response to distinct physiological and energetic environments.

In contrast to FliJ, *Vibrio* FliH and FliI exhibited clear interspecies incompatibility when expressed individually in *Salmonella*. Pull-down assays demonstrated that FliH-FliI interactions are strictly species-specific: *Vibrio* FliH interacted with *Vibrio* FliI but not with *Salmonella* FliI, and vice versa (Figure 4). These findings indicate that species-specific physical interactions between FliH and FliI underlie the observed functional incompatibility. Although overall folds of FliH and FliI are conserved (Supplementary Figures 1B, 2B), subtle differences at their interaction interfaces likely prevent the formation of stable heterologous complexes. Thus, unlike FliJ, the FliH-FliI module appears to be subject to tighter evolutionary tuning, limiting its functional interchangeability across species.

Our ion-dependence analyses provide direct experimental evidence that the two major functions of the flagellar ATPase complex—substrate delivery and export gate activation—are mechanistically separable. In the *Salmonella* Δ*fliH-fliI* mutant expressing *Vibrio* FliH and FliI, FlgD export occurred only in the presence of Na^+^, a condition known to support protein export when the ATPase complex is non-functional. This phenotype indicates that the *Vibrio* FliH-FliI complex can function as the dynamic carrier that delivers substrates to the export gate but is insufficient to activate the H^+^-driven transport mode. By contrast, the native *Salmonella* ATPase complex supports efficient export independently of Na^+^. These results demonstrate that the ATP hydrolysis-driven gate activation represents a specialized function that is uncoupled from substrate delivery.

High-speed atomic force microscopy revealed that the *Vibrio* FliH-FliI complex assembles into a ring-shaped structure that closely resembles that formed by the *Salmonella* FliH-FliI complex (Figure 6). In addition, biochemical cross-linking experiments showed that *Vibrio* FliH forms a dimer and that the FliH-FliI complex assembles into a heterotrimeric unit, consistent with the canonical architecture of the ATPase complex (Figure 5B). These observations rule out gross defects in complex assembly as the primary cause of impaired gate activation. Instead, they suggest that dynamic or transient interactions between the ATPase complex and the export gate, rather than static structural features, are critical for coupling ATP hydrolysis to H^+^-driven protein transport.

ATP hydrolysis by the FliI ATPase is required for efficient and robust gate activation via specific interactions between FliJ and FlhA (Minamino et al., 2011; Minamino et al., 2014). *Vibrio* FliJ requires *Salmonella* FliH and FliI to efficiently serve as an activator of the H^+^-driven export engine in the *Salmonella* fT3SS export gate. Based on our results, we propose a model in which FliJ functions as a universally conserved activator of the export gate, while the FliH-FliI complex serves as a species-specific regulator that fine-tunes the interaction between FliJ and FlhA, thereby enabling the export gate to become an active H^+^-driven protein transporter. The cytoplasmic ATPase ring complex is anchored to the basal body C-ring through interactions between the extreme N-terminal region of FliH, including Trp-7 and Trp-10, and FliN (Minamino et al., 2009; Hara et al., 2012; Notti et al., 2015). Although *Vibrio* FliH and FliI assembled into a ring structure (Figure 6B), they failed to activate the H^+^-driven export engine (Figure 3B). Notably, the N-terminal region of *Vibrio* FliH is 29 residues longer than that of *Salmonella* FliH (Supplementary Figure 1A), suggesting that proper FliH-FliN interactions are additionally required for precise functional coupling between ATP hydrolysis by the ATPase complex and gate activation. Future structural and biophysical studies focusing on the dynamic interactions among the FliH-FliI complex, FliJ, and FlhA will be essential to elucidate the molecular basis of this coupling. Together, our study provides a conceptual framework for understanding how the flagellar ATPase complex has evolved to integrate environmental ion availability with efficient and ordered protein export. More broadly, our findings suggest that functional specialization within conserved molecular machines can arise from divergence in dynamic coupling mechanisms rather than from changes in core architecture.

These findings are consistent with previous structural studies showing that export chaperone-substrate complexes interact with the FlhA platform prior to secretion (Xing et al., 2018), and that FliJ can associate with chaperone complexes (Rossi et al., 2023). In addition, structural analyses of homologous type III ATPases, such as the EscN hexamer (Majewski et al., 2019), have provided important insights into the conserved architecture and function of ATPase assemblies, supporting the relevance of our observations.

The Na^+^-driven flagellar motor in marine *Vibrio* is known to exhibit higher rotational speed than the H^+^-driven motor in *Salmonella* (Magariyama et al., 1994), likely reflecting differences in ion flux rates and stator unit dynamics. Our findings raise the possibility that similar principles may apply to the export gate, where Na^+^-coupled protein export is favored under conditions in which ATPase-mediated activation is compromised. This suggests that the dual-fuel capability of the export gate provides functional flexibility to adapt to different energetic environments.

Despite these advances, how substrate delivery by the ATPase complex is mechanistically coupled to export gate activation remains unclear. Beyond its mechanistic implications, our findings may have relevance for bacterial pathogenicity and the development of alternative antimicrobial strategies. Flagellar assembly and motility contribute critically to host colonization, tissue invasion, and biofilm formation in many pathogenic bacteria. The fT3SS therefore represents a key regulatory node in virulence rather than in bacterial viability. Our demonstration that substrate delivery and export gate activation are mechanistically separable identifies the ATPase-gate coupling as a previously unrecognized vulnerability in the flagellar export apparatus. Importantly, disruption of this coupling without abolishing ATPase assembly itself may selectively impair flagellar biogenesis and virulence while imposing reduced selective pressure for resistance compared with conventional bactericidal antibiotics. Thus, the species-specific tuning of ATPase-gate interactions revealed here would provide a conceptual framework for targeting energy-coupling mechanisms of bacterial secretion systems as anti-virulence strategies, opening new avenues for antimicrobial intervention that exploit biophysical constraints rather than essential metabolic functions.

## Data Availability

The original contributions presented in the study are included in the article/Supplementary material, further inquiries can be directed to the corresponding author/s.

## Footnotes

1
http://www.ebi.ac.uk/Tools/msa/clustalo/

2
https://alphafoldserver.com

## References

[ref1] Abrusci P. Vergara-Irigaray M. Johnson S. Beeby M. D. Hendrixson D. R. Roversi P. . (2013). Architecture of the major component of the type III secretion system export apparatus. Nat. Struct. Mol. Biol. 20, 99–104. doi: 10.1038/nsmb.2452, 23222644 PMC3537844

[ref2] Ando T. Kodera N. Takai E. Maruyama D. Saito S. Toda A. (2001). A high-speed atomic force microscope for studying biological macromolecules. Proc. Natl. Acad. Sci. USA 98, 12468–12472. doi: 10.1073/pnas.211400898, 11592975 PMC60077

[ref3] Ando T. Uchihashi T. Fukuma T. (2008). High-speed atomic force microscopy for nano-visualization of dynamic biomolecular processes. Prog. Surf. Sci. 83, 337–437. doi: 10.1016/j.progsurf.2008.09.001

[ref4] Auvray F. Ozin A. J. Claret L. Hughes C. (2002). Intrinsic membrane targeting of the flagellar export ATPase FliI: interaction with acidic phospholipids and FliH. J. Mol. Biol. 318, 941–950. doi: 10.1016/S0022-2836(02)00172-9, 12054792 PMC2528292

[ref5] Bai F. Morimoto Y.V. Yoshimura S.D.J. Hara N. Kami-Ike N. Namba K (2014). Assembly dynamics and the roles of FliI ATPase of the bacterial flagellar export apparatus. Sci. Rep. 4:6528. doi: 10.1038/srep06528 25284201 PMC4185386

[ref6] Bange G. Kümmerer N. Engel C. Bozkurt G. Wild K. Sinning I. (2010). FlhA provides the adaptor for coordinated delivery of late flagella building blocks to the type III secretion system. Proc. Natl. Acad. Sci. USA 107, 11295–11300. doi: 10.1073/pnas.1001383107, 20534509 PMC2895114

[ref7] Claret L. Calder S. R. Higgins M. Hughes C. (2003). Oligomerization and activation of the FliI ATPase central to bacterial flagellum assembly. Mol. Microbiol. 48, 1349–1355. doi: 10.1046/j.1365-2958.2003.03506.x, 12787361 PMC2528289

[ref8] González-Pedrajo B. Fraser G. M. Minamino T. Macnab R. M. (2002). Molecular dissection of *Salmonella* FliH, a regulator of the ATPase FliI and the type III flagellar protein export pathway. Mol. Microbiol. 45, 967–982. doi: 10.1046/j.1365-2958.2002.03047.x, 12180917

[ref9] González-Pedrajo B. Minamino T. Kihara M. Namba K. (2006). Interactions between C ring proteins and export apparatus components: a possible mechanism for facilitating type III protein export. Mol. Microbiol. 60, 984–998. doi: 10.1111/j.1365-2958.2006.05149.x, 16677309

[ref10] Hara N. Morimoto Y. V. Kawamoto A. Namba K. Minamino T. (2012). Interaction of the extreme N-terminal region of FliH with FlhA is required for efficient bacterial flagellar protein export. J. Bacteriol. 194, 5353–5360. doi: 10.1128/JB.01028-12, 22843851 PMC3457192

[ref11] Ibuki T. Imada K. Minamino T. Kato T. Miyata T. Namba K. (2011). Common architecture between the flagellar protein export apparatus and F- and V-ATPases. Nat. Struct. Mol. Biol. 18, 277–282. doi: 10.1038/nsmb.1977, 21278755

[ref12] Ibuki T. Uchida Y. Hironaka Y. Namba K. Imada K. Minamino T. (2013). Interaction between FliJ and FlhA, components of the bacterial flagellar type III export apparatus. J. Bacteriol. 195, 466–473. doi: 10.1128/JB.01711-12, 23161028 PMC3554004

[ref13] Imada K. Minamino T. Kinoshita M. Furukawa Y. Namba K. (2010). Structural insight into the regulatory mechanisms of interactions of the flagellar type III chaperone FliT with its binding partners. Proc. Natl. Acad. Sci. USA 107, 8812–8817. doi: 10.1073/pnas.1001866107, 20421493 PMC2889304

[ref14] Imada K. Minamino T. Tahara A. Namba K. (2007). Structural similarity between the flagellar type III ATPase FliI and F1-ATPase subunits. Proc. Natl. Acad. Sci. USA 104, 485–490. doi: 10.1073/pnas.0608090104, 17202259 PMC1766411

[ref15] Imada K. Minamino T. Uchida Y. Kinoshita M. Namba K. (2016). Insight into the flagella type III export revealed by the complex structure of the type III ATPase and its regulator. Proc. Natl. Acad. Sci. USA 113, 3633–3638. doi: 10.1073/pnas.1524025113, 26984495 PMC4822572

[ref16] Inoue Y. Morimoto Y. V. Namba K. Minamino T. (2018). Novel insights into the mechanism of well-ordered assembly of bacterial flagellar proteins in *Salmonella*. Sci. Rep. 8:1787. doi: 10.1038/s41598-018-20209-3, 29379125 PMC5789064

[ref17] Johnson S. Furlong E. J. Deme J. C. Nord A. L. Caesar J. J. E. Chevance F. F. V. . (2021). Molecular structure of the intact bacterial flagellar basal body. Nat. Microbiol. 6, 712–721. doi: 10.1038/s41564-021-00895-y, 33931760 PMC7610862

[ref18] Kazetani K. Minamino T. Miyata T. Kato T. Namba K. (2009). ATP-induced FliI hexamerization facilitates bacterial flagellar protein export. Biochem. Biophys. Res. Commun. 388, 323–327. doi: 10.1016/j.bbrc.2009.08.004, 19665005

[ref19] Kinoshita M. Hara N. Imada K. Namba K. Minamino T. (2013). Interactions of bacterial chaperone-substrate complexes with FlhA contribute to coordinating assembly of the flagellar filament. Mol. Microbiol. 90, 1249–1261. doi: 10.1111/mmi.12430, 24325251

[ref1101] Kinoshita M. Namba K. Minamino T. (2021). A positive charge region of *Salmonella* FliI is required for ATPase formation and efficient flagellar protein export. Commun. Biol. 4:464. doi: 10.1038/s42003-021-01980-y 33846530 PMC8041783

[ref20] Kinoshita M. Minamino T. Uchihashi T. Namba K. (2024). FliH and FliI help FlhA bring strict order to flagellar protein export in *Salmonella*. Commun. Biol. 7:366. doi: 10.1038/s42003-024-06081-0, 38531947 PMC10965912

[ref21] Kinoshita M. Miyata T. Makino F. Imada K. Namba K. Minamino T. (2025). A β-cap on the FliPQR protein-export channel acts as the cap for initial flagellar rod assembly. Proc. Natl. Acad. Sci. USA 122:e2507221122. doi: 10.1073/pnas.2507221122, 40833400 PMC12403130

[ref22] Kuhlen L. Abrusci P. Johnson S. Gault J. Deme J. Caesar J. . (2018). Structure of the core of the type III secretion system export apparatus. Nat. Struct. Mol. Biol. 25, 583–590. doi: 10.1038/s41594-018-0086-9, 29967543 PMC6233869

[ref23] Kuhlen L. Johnson S. Zeitler A. Bäurle S. Deme J. C. Caesar J. J. E. . (2020). The substrate specificity switch FlhB assembles onto the export gate to regulate type three secretion. Nat. Commun. 11:1296. doi: 10.1038/s41467-020-15071-9, 32157081 PMC7064499

[ref24] Magariyama Y. Sugiyama S. Muramoto K. Maekawa Y. Kawagishi I. Imae Y. . (1994). Very fast flagellar rotation. Nature 371:752. doi: 10.1038/371752b0, 7935835

[ref25] Majewski D. D. Worrall L. J. Hong C. Atkinson C. E. Vuckovic M. Watanabe N. . (2019). Cryo-EM structure of the homohexameric T3SS ATPase-central stalk complex reveals rotary ATPase-like asymmetry. Nat. Commun. 10:626. doi: 10.1038/s41467-019-08477-7, 30733444 PMC6367419

[ref26] McMurry J. L. Murphy J. W. González-Pedrajo B. (2006). The FliN-FliH interaction mediates localization of flagellar export ATPase FliI to the C ring complex. Biochemistry 45, 11790–11798. doi: 10.1021/bi0605890, 17002279

[ref27] Minamino T. Chu R. Yamaguchi S. Macnab R. M. (2000). Role of FliJ in flagellar protein export in *Salmonella*. J. Bacteriol. 182, 4207–4215. doi: 10.1128/JB.182.15.4207-4215.2000, 10894728 PMC101910

[ref28] Minamino T. González-Pedrajo B. Kihara M. Namba K. Macnab R. M. (2003). The ATPase FliI can interact with the type III flagellar protein export apparatus in the absence of its regulator, FliH. J. Bacteriol. 185, 3983–3988. doi: 10.1128/JB.185.13.3983-3988.2003, 12813095 PMC161568

[ref29] Minamino T. Kazetani K. Tahara A. Suzuki H. Furukawa Y. Kihara M. . (2006). Oligomerization of the bacterial flagellar ATPase FliI is controlled by its extreme N-terminal region. J. Mol. Biol. 360, 510–519. doi: 10.1016/j.jmb.2006.05.010, 16780875

[ref30] Minamino T. Kinoshira M. Imada K. Namba K. (2012). Interaction between FliI ATPase and a flagellar chaperone FliT during bacterial flagellar export. Mol. Microbiol. 83, 168–178. doi: 10.1111/j.1365-2958.2011.07924.x, 22111876

[ref31] Minamino T. Kinoshita M. (2023). Structure, assembly, and function of flagella responsible for bacterial locomotion. EcoSal Plus 11:eesp-0011-2023. doi: 10.10.1128/ecosalplus.esp-0011-2023 PMC10729930 37260402

[ref32] Minamino T. Kinoshita M. Inoue Y. Morimoto Y. V. Ihara K. Koya S. . (2016a). FliH and FliI ensure efficient energy coupling of flagellar type III protein export in *Salmonella*. MicrobiologyOpen. 5, 424–435. doi: 10.1002/mbo3.340, 26916245 PMC4905995

[ref33] Minamino T. Kinoshita M. Morimoto Y.V. Namba K. (2021a). The FlgN chaperone activates the Na^+^-driven engine of the Salmonella flagellar protein export apparatus. Commun. Biol. 4:335. doi: 10.1038/s42003-021-01865-0 33712678 PMC7955116

[ref34] Minamino T. Kinoshita M. Morimoto Y. V. Namba K. (2022a). Activation mechanism of the bacterial flagellar dual-fuel protein export engine. Biophys. Physicobiol. 19:e190046. doi: 10.2142/biophysico.bppb-v19.0046, 36567733 PMC9751260

[ref35] Minamino T. Kinoshita M. Namba K. (2022b). Insight into distinct functional roles of the flagellar ATPase complex for flagellar assembly in Salmonella. Front. Microbiol. 13:864178. doi: 10.3389/fmicb.2022.864178, 35602071 PMC9114704

[ref36] Minamino T. Macnab R. M. (2000a). FliH, a soluble component of the type III flagellar export apparatus of *Salmonella*, forms a complex with FliI and inhibits its ATPase activity. Mol. Microbiol. 37, 1494–1503. doi: 10.1046/j.1365-2958.2000.02106.x, 10998179

[ref37] Minamino T. Macnab R. M. (2000b). Interactions among components of the *Salmonella* flagellar export apparatus and its substrates. Mol. Microbiol. 35, 1052–1064. doi: 10.1046/j.1365-2958.2000.01771.x, 10712687

[ref38] Minamino T. Morimoto Y. V. Hara N. Aldridge P. D. Namba K. (2016b). The bacterial flagellar type III export gate complex is a dual fuel engine that can use both H^+^ and Na^+^ for flagellar protein export. PLoS Pathog. 12:e1005495. doi: 10.1371/journal.ppat.1005495, 26943926 PMC4778876

[ref39] Minamino T. Morimoto Y. V. Hara N. Namba K. (2011). An energy transduction mechanism used in bacterial flagellar type III protein export. Nat. Commun. 2:475. doi: 10.1038/ncomms1488, 21934659 PMC3195256

[ref40] Minamino T. Morimoto Y. V. Kinoshita M. Aldridge P. D. Namba K. (2014). The bacterial flagellar protein export apparatus processively transports flagellar proteins even with extremely infrequent ATP hydrolysis. Sci. Rep. 4:7579. doi: 10.1038/srep07579, 25531309 PMC4273619

[ref41] Minamino T. Morimoto Y. V. Kinoshita M. Namba K. (2021b). Membrane voltage-dependent activation mechanism of the bacterial flagellar protein export apparatus. Proc. Natl. Acad. Sci. USA 118:e2026587118. doi: 10.1073/pnas.2026587118, 34035173 PMC8179193

[ref42] Minamino T. Namba K. (2008). Distinct roles of the FliI ATPase and proton motive force in bacterial flagellar protein export. Nature 451, 485–488. doi: 10.1038/nature06449, 18216858

[ref43] Minamino T. Yoshimura S. D. J. Morimoto Y. V. González-Pedrajo B. Kami-Ike N. Namba K. (2009). Roles of the extreme N-terminal region of FliH for efficient localization of the FliH-FliI complex to the bacterial flagellar type III export apparatus. Mol. Microbiol. 74, 1471–1483. doi: 10.1111/j.1365-2958.2009.06946.x, 19889085

[ref44] Morimoto Y. V. Ito M. Hiraoka K. D. Che Y. S. Bai F. Kami-Ike N. . (2014). Assembly and stoichiometry of FliF and FlhA in *Salmonella* flagellar basal body. Mol. Microbiol. 91, 1214–1226. doi: 10.1111/mmi.12529, 24450479

[ref45] Notti R. Q. Bhattacharya S. Lilic M. Stebbins C. E. (2015). A common assembly module in injectisome and flagellar type III secretion sorting platforms. Nat. Commun. 6:7125. doi: 10.1038/ncomms8125, 25994170 PMC4443714

[ref46] Ohnishi K. Fan F. Schoenhals G. J. Kihara M. Macnab R. M. (1997). The FliO, FliP, FliQ, and FliR proteins of *Salmonella typhimurium*: putative components for flagellar assembly. J. Bacteriol. 179, 6092–6099. doi: 10.1128/jb.179.19.6092-6099.1997, 9324257 PMC179513

[ref47] Ohnishi K. Ohto Y. Aizawa S. Macnab R. M. Iino T. (1994). FlgD is a scaffolding protein needed for flagellar hook assembly in *Salmonella typhimurium*. J. Bacteriol. 176, 2272–2281. doi: 10.1128/jb.176.8.2272-2281.1994, 8157595 PMC205349

[ref48] Paul K. Erhardt M. Hirano T. Blair D. F. Hughes K. T. (2008). Energy source of flagellar type III secretion. Nature 451, 489–492. doi: 10.1038/nature06497, 18216859

[ref49] Paul K. Harmon J. G. Blair D. F. (2006). Mutational analysis of the flagellar rotor protein FliN: identification of surfaces important for flagellar assembly and switching. J. Bacteriol. 188, 5240–5248. doi: 10.1128/JB.00110-06, 16816196 PMC1539977

[ref50] Pettersen E. F. Goddard T. D. Huang C. C. Meng E. C. Couch G. S. Croll T. I. . (2021). UCSF ChimeraX: structure visualization for researchers, educators, and developers. Protein Sci. 30, 70–82. doi: 10.1002/pro.3943, 32881101 PMC7737788

[ref51] Rossi P. Xing Q. Bini E. Portaliou A. G. Clay M. C. Warren E. M. . (2023). Chaperone recycling in late-stage flagellar assembly. J. Mol. Biol. 435:167954. doi: 10.1016/j.jmb.2023.167954, 37330284 PMC10471782

[ref52] Thomas J. Stafford G. P. Hughes C. (2004). Docking of cytosolic chaperone-substrate complexes at the membrane ATPase during flagellar type III protein export. Proc. Natl. Acad. Sci. USA 101, 3945–3950. doi: 10.1073/pnas.0307223101, 15001708 PMC374349

[ref53] Wagner S. Diepold A. (2020). A unified nomenclature for injectisome-type type III secretion systems. Curr. Top. Microbiol. Immunol. 427, 1–10. doi: 10.1007/82_2020_210, 32415388

[ref54] Xing Q. Shi K. Portaliou A. Rossi P. Economou A. Kalodimos C. G. (2018). Structure of chaperone-substrate complexes docked onto the export gate in a type III secretion system. Nat. Commun. 9:1773. doi: 10.1038/s41467-018-04137-4, 29720631 PMC5932034

