# Supplementary material for: Uncoupling substrate delivery from export gate activation reveals distinct roles of the flagellar ATPase complex
Source: Front Microbiol. 2026 May 21;17:1841110. doi: 10.3389/fmicb.2026.1841110 (PMC13233425; doi:10.3389/fmicb.2026.1841110)
Supplement: Supplementary file 1 [file Data_Sheet_1.pdf]

Supplementary Information

Uncoupling substrate delivery from export gate activation reveals distinct roles of the flagellar ATPase complex

**Tohru Minamino, Miki Kinoshita, Yuki Tajimi,
Takayuki Uchihashi, and Keiichi Namba**

A

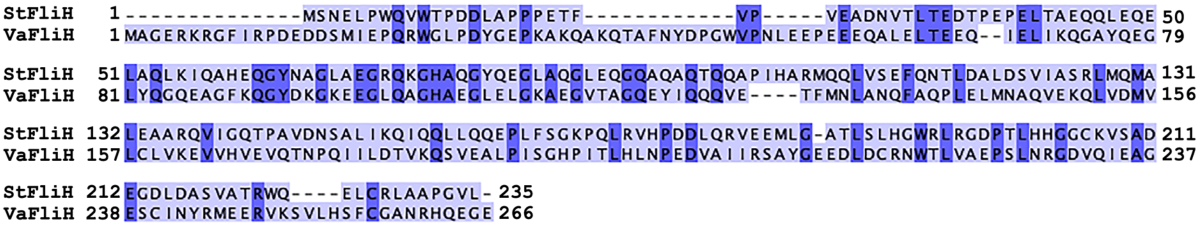

B

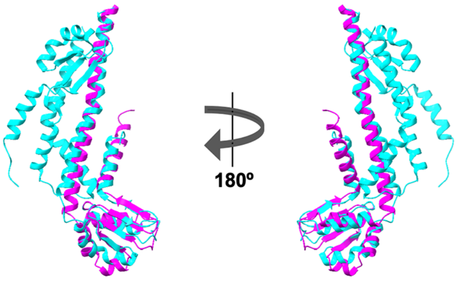

StFliH$_{C2}$, cyan; VaFliH$_{C}$, magenta

Supplementary Figure 1. Sequence and structural comparison of *Salmonella* and *Vibrio* FliH. (A) Pairwise sequence alignment of FliH proteins of *Salmonella enterica* serovar Typhimurium (StFliH) and *Vibrio alginolyticus* (VaFliH). Sequence alignment was carried out by Clustal Omega. (B) Structural comparison of the crystal structure of the C-terminal domains of the *Salmonella* FliH dimer (FliH$_{C2}$, cyan) (PDB ID: 5B0O) and AlphaFold predicted structure of the C-terminal domain of *Vibrio* FliH (residues 124–266) (VaFliH$_{C}$, magenta) using ChimeraX.

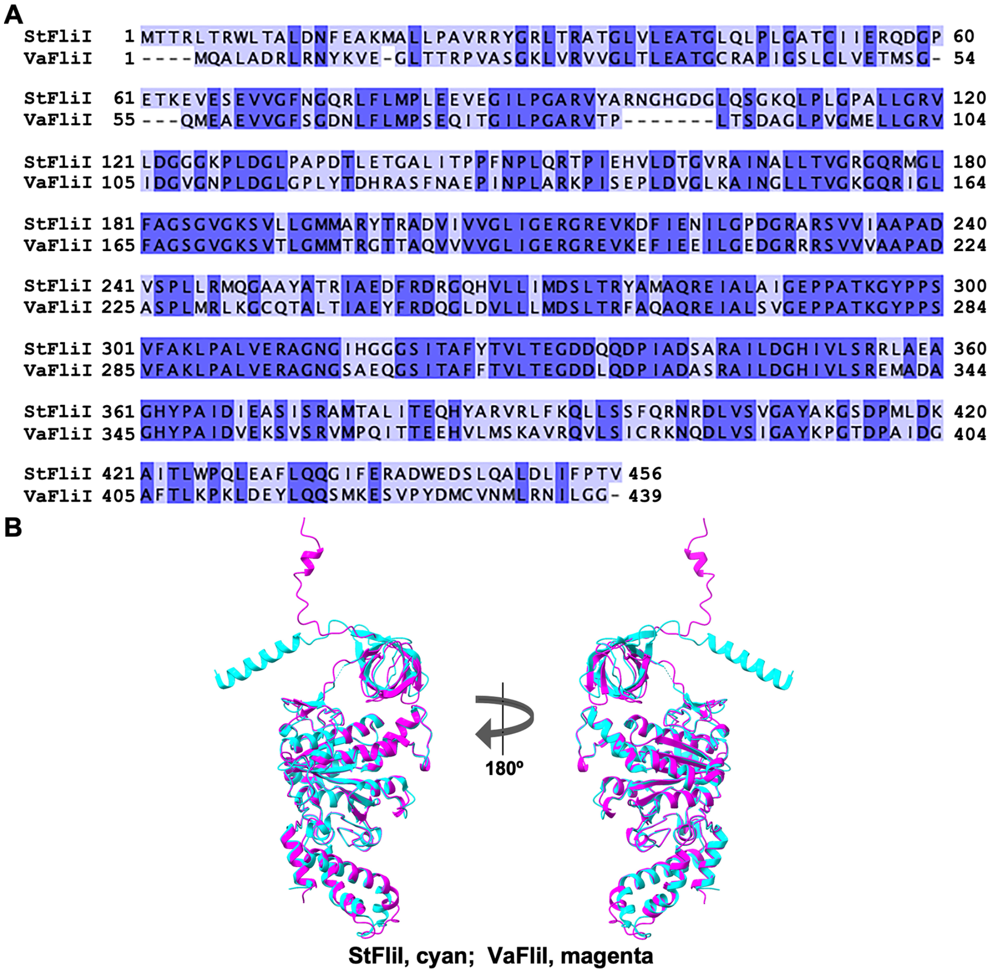

Supplementary Figure 2. Sequence and structural comparison of *Salmonella* and *Vibrio* FliI. (A) Pairwise sequence alignment of FliI proteins of *Salmonella enterica* serovar Typhimurium (StFliI) and *Vibrio alginolyticus* (VaFliI). Sequence alignment was carried out by Clustal Omega. (B) Structural comparison of *Salmonella* FliI crystal structure (cyan) (PDB ID: 5B0O) and AlphaFold predicted structure of *Vibrio* FliI (magenta) using ChimeraX.

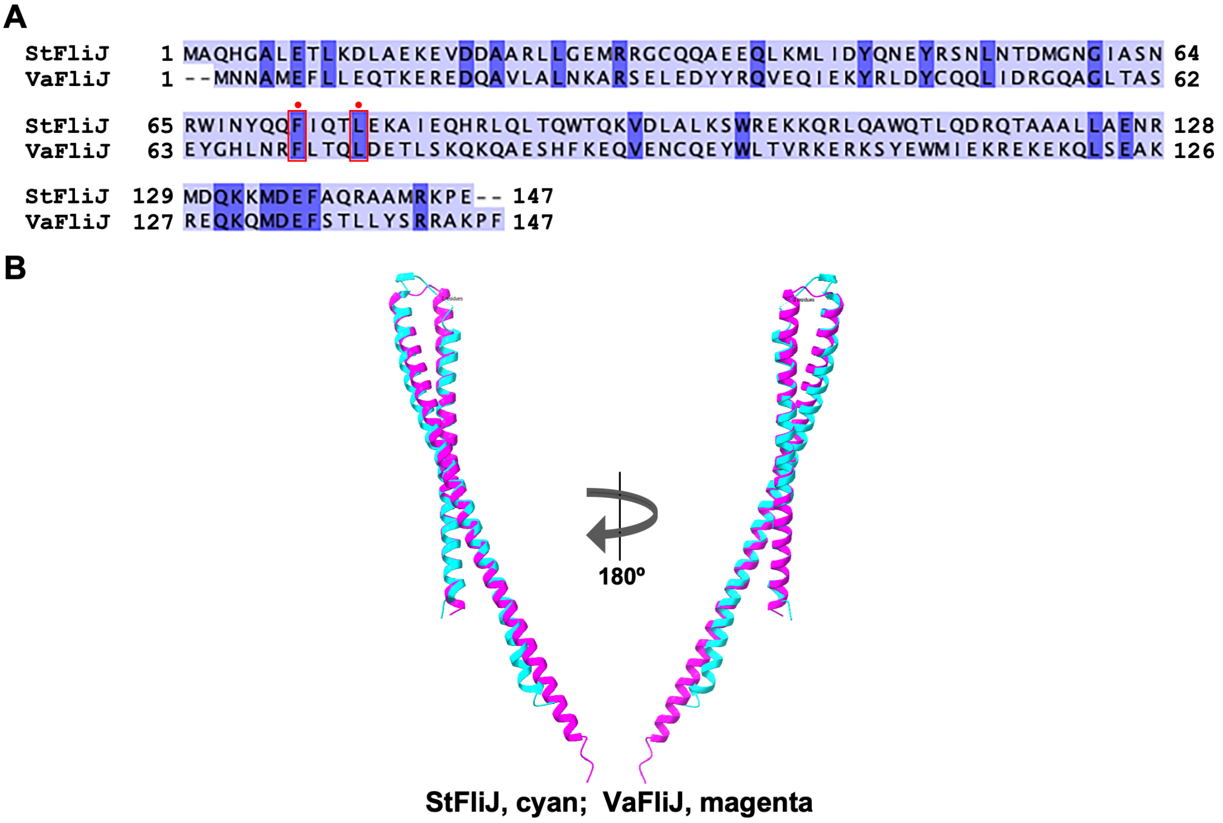

Supplementary Figure 3. Sequence and structural comparison of *Salmonella* and *Vibrio* FliJ. (A) Pairwise sequence alignment of FliJ proteins of *Salmonella enterica* serovar Typhimurium (StFliJ) and *Vibrio alginolyticus* (VaFliJ). Sequence alignment was carried out by Clustal Omega. The two highly conserved residues of FliJ, Phe-72 and Leu-76, indicated by red dots, have been reported to be directly involved in the interaction with FlhA. (B) Structural comparison of *Salmonella* FliJ crystal structure (cyan) (PDB ID: 3AJW) and AlphaFold predicted structure of *Vibrio* FliJ (magenta) using ChimeraX.

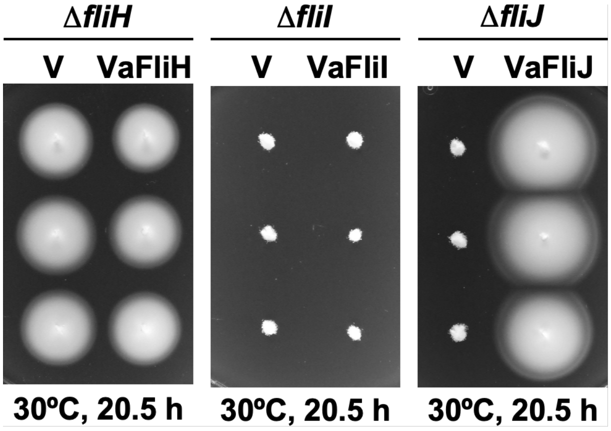

Supplementary Figure 4. Cross-complementation analysis of *Vibrio* ATPase components in *Salmonella*. (A) Soft-agar motility assays of a *Salmonella enterica* serovar Typhimurium Δ*fliH,* Δ*fliI, and* Δ*fliJ* mutants expressing *Vibrio alginolyticus* FliH (VaFliH), FliI (VaFliI), or FliJ (VaFliJ) individually. pTrc99AFF4 was used as a vector control (V). Plates were incubated at 30°C for 20.5 h. At least seven independent assays were performed.

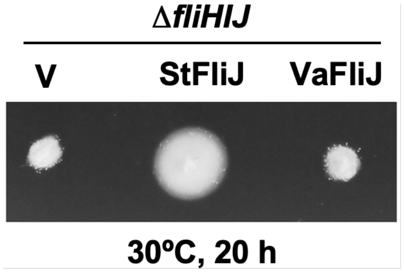

Supplementary Figure 5. Effect of deletion of native FliH and FliI on motility of the *Salmonella* Δ*fliJ* mutant expressing *Vibrio* FliJ. Soft-agar motility assays of a *Salmonella enterica* serovar Typhimurium Δ*fliH-fliI-fliJ* mutant (Δ*fliHIJ*) carrying pTrc99AFF4 (V), pMM404 (StFliJ), or pMKM2003Va (VaFliJ). Plates were incubated at 30°C for 20.5 h. At least seven independent assays were performed.

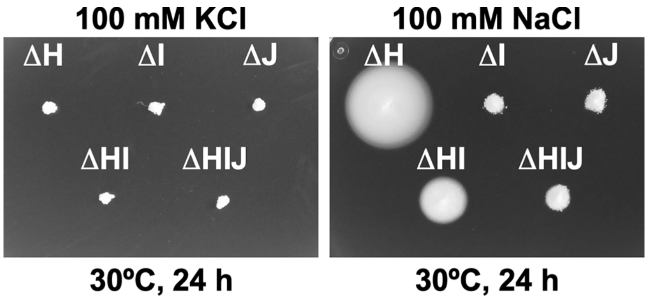

Supplementary Figure 6. Effect of sodium ions on motility of the *Salmonella* mutant lacking the flagellar ATPase components. Soft-agar motility assays of *Salmonella enterica* serovar Typhimurium Δ*fliH* (ΔH), Δ*fliI* (ΔI), Δ*fliJ* (ΔJ), Δ*fliH-fliI* (ΔHI), and Δ*fliH-fliI-fliJ* (Δ*fliHIJ*) mutants. Plates were incubated at 30°C for 24 h. At least seven independent assays were performed.

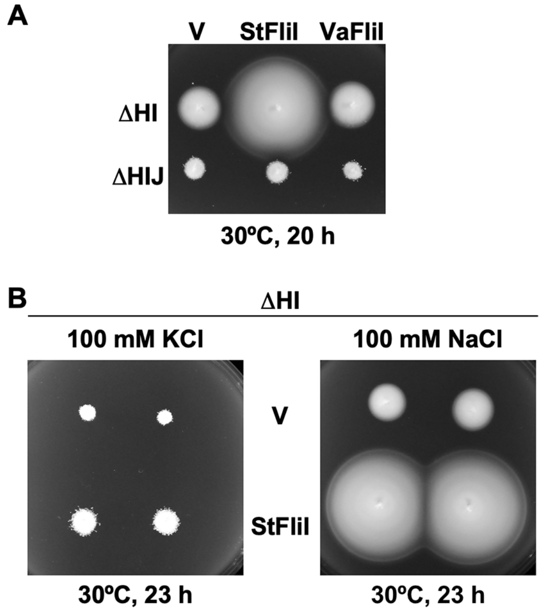

Supplementary Figure 7. Multicopy effect of FliI on motility of *Salmonella* mutants lacking the flagellar ATPase components. (A) Soft-agar motility assays of *Salmonella enterica* serovar Typhimurium Δ*fliH-fliI* (ΔHI) and Δ*fliH-fliI-fliJ* (ΔHIJ) mutants carrying pTrc99A (V), pMM1702 (StFliI), or pMKM1702Va (VaFliI). Plates were incubated at 30°C for 20 h. At least seven independent assays were performed. (B) Motility of the *Salmonella* Δ*fliH-fliI* mutant carrying pTrc99A or pMM1702 on soft agar plates supplemented with either 100 mM KCl (K^+) or 100 mM NaCl (Na^+). Plates were incubated at 30°C for 23 h.

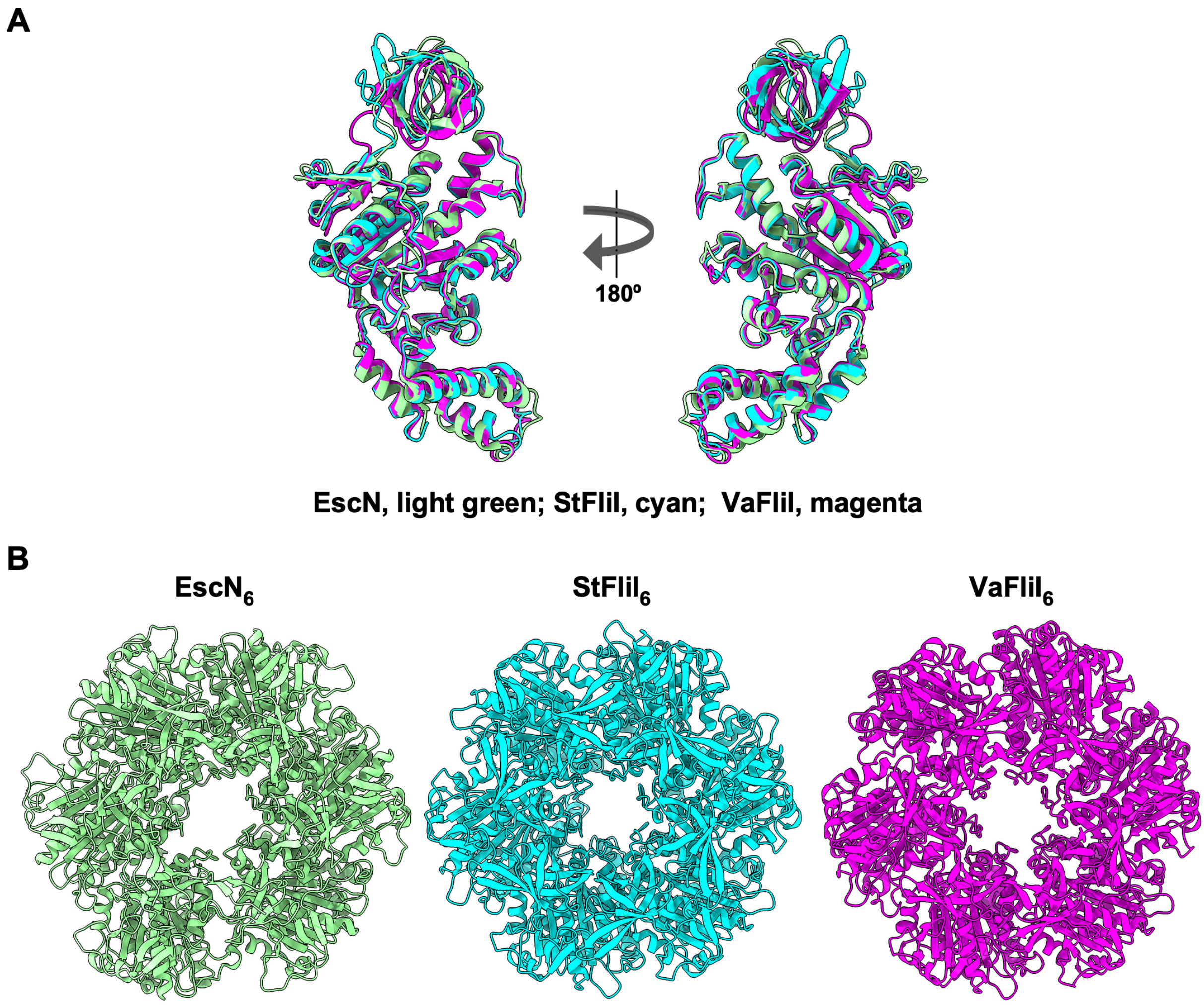

Supplementary Figure 8. AlphaFold-predicted structures of FliI homohexamers from *Salmonella enterica* serovar Typhimurium ($StFliI_6$) and *Vibrio alginolyticus* ($VaFliI_6$). (A) Superposition of the AlphaFold-predicted structures of *Salmonella* FliI (cyan) and *Vibrio* FliI (magenta) onto the cryo-EM structure of EscN (light green, PDB ID: 6NJO) using ChimeraX. (B) Structural comparison of the EscN homohexamer with the AlphaFold-predicted FliI homohexamers.

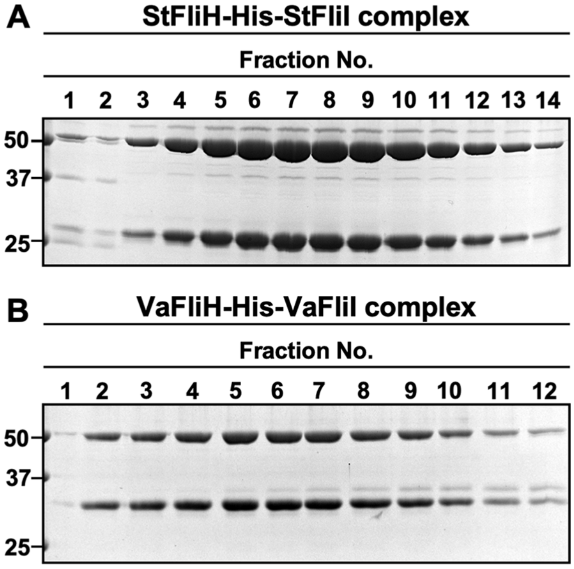

Supplementary Figure 9. Purification of the FliH-FliI complexes by size exclusion chromatography. The FliH-His-FliI complexes from (A) *Salmonella enterica* serovar Typhimurium and (B) *Vibrio alginolyticus*, purified by Ni-affinity chromatography, were applied to a Superdex 200 16/60 column equilibrated with buffer containing 50 mM Tris-HCl (pH 8.0), 150 mM NaCl and 1 mM EDTA at a flow rate of 1.0 ml/min. Elution fractions were analyzed by SDS-PAGE, followed by Coomassie Brilliant Blue staining. Positions of molecular mass markers (kDa) are indicated on the left.
